# A Comprehensive Review of Congenital Eye Diseases in Pediatrics: Etiology, Diagnosis, and Management

**DOI:** 10.7759/cureus.92753

**Published:** 2025-09-19

**Authors:** Sara M Samhan, Sultan A Alqasim, Alreem S Aldwsri, Maram A Alhazmi, Raghad I Azzoni, Lara H Mugharbel, Shaden M Hazmi, Rund A Almohaish, Seham H Zaebi, Baraa Alghalyini, Abdul Rehman Z Zaidi

**Affiliations:** 1 College of Medicine, Alfaisal University, Riyadh, SAU; 2 College of Medicine, King Saud bin Abdulaziz University for Health Sciences, Riyadh, SAU; 3 College of Medicine, Arabian Gulf University, Manama, BHR; 4 College of Medicine, Taibah University, Medina, SAU; 5 College of Medicine, King Faisal University, Al-Ahsa, SAU; 6 College of Medicine, Jazan University, Jazan, SAU; 7 Department of Family and Community Medicine, Alfaisal University, Riyadh, SAU

**Keywords:** childhood blindness, congenital cataracts, congenital eye diseases, genetic etiology, macular dystrophies, microphthalmia, pediatric ophthalmology, retinal dystrophies, retinopathy of prematurity (rop), vision impairment in children

## Abstract

Congenital eye diseases in children, arising from genetic mutations and developmental anomalies, are a significant cause of childhood visual impairment worldwide and often lead to lifelong visual disability if left untreated. This narrative review synthesizes current knowledge and emerging evidence on etiology, including genetic and environmental factors, clinical manifestations, diagnostic approaches, and management strategies for key pediatric ocular conditions such as congenital cataracts, retinopathy of prematurity (ROP), inherited retinal dystrophies, macular dystrophies, and microphthalmia. Genetic underpinnings are a common theme, with many of these disorders being associated with identifiable gene mutations and emerging gene therapies offering hope for conditions previously deemed untreatable. Contemporary diagnostic tools such as advanced ocular imaging and genetic testing facilitate early detection, which is critical for improved prognoses. Therapeutic advances are highlighted, from refined surgical techniques (e.g., early cataract extraction) to novel interventions like gene replacement therapy for inherited retinal dystrophies and stem cell applications aimed at retinal regeneration. Despite these advances, significant research gaps remain, particularly in understanding long-term outcomes, improving treatment accessibility for rare diseases, and translating emerging therapies into clinical practice. Emphasis is placed on early detection via screening programs (e.g., newborn eye exams, ROP screening) and timely multidisciplinary interventions, which are crucial for improving visual outcomes.

## Introduction and background

Congenital eye diseases are a diverse group of ocular anomalies present at birth, significantly impacting vision and overall quality of life in affected children. These conditions range from structural abnormalities to functional impairments, encompassing congenital cataracts, retinopathy of prematurity (ROP), retinal dystrophies, macular dystrophies, and microphthalmia. Globally, congenital eye diseases are among the leading causes of childhood blindness, contributing to approximately 1.4 million cases of visual impairment in children. The burden of these diseases is particularly pronounced in low- and middle-income countries due to limited access to early diagnosis and intervention services [1].

The etiology of congenital eye diseases is multifaceted, involving genetic mutations, intrauterine infections, and environmental factors. Genetic disorders contribute to 11-39% of childhood blindness, with a higher prevalence observed in developed countries [2]. Intrauterine infections, such as rubella and toxoplasmosis, have been implicated in the development of congenital ocular anomalies [3]. Additionally, socioeconomic determinants, including limited access to prenatal care and early intervention services, exacerbate the burden of these diseases in underserved populations [4].

The public health implications of congenital eye diseases extend beyond visual impairment to encompass broader societal and economic challenges. In the United States alone, the economic burden of childhood vision disorders is estimated at $10 billion annually [5]. Early detection and timely intervention are crucial for improving visual outcomes and enhancing the quality of life of affected children. Despite advances in diagnostic modalities and therapeutic approaches, significant disparities in access to care persist, particularly in low-resource settings [6].

This review provides a comprehensive analysis of five major congenital eye diseases: congenital cataracts, ROP, retinal dystrophies, macular dystrophies, and microphthalmia. It explores their epidemiology, etiological factors, clinical manifestations, diagnostic approaches, and current therapeutic interventions. Additionally, this review highlights emerging trends in gene therapy, stem cell applications, and personalized medicine while identifying research gaps and future directions. By synthesizing the latest evidence, this review aims to inform clinical practice and guide public health policy to address the multifaceted challenges posed by congenital eye diseases in pediatric populations.

## Review

Methods

This review was conducted as a narrative literature synthesis, following established guidelines for narrative reviews to ensure rigor and transparency. In particular, we adhered to the principles of the Scale for the Assessment of Narrative Review Articles (SANRA) framework, which promotes a structured and transparent approach to narrative reviews​ [7]. The scope of the review was defined to cover major congenital eye diseases in pediatric populations, specifically congenital cataracts, ROP, inherited retinal dystrophies, developmental macular dystrophies, and microphthalmia/anophthalmia. Below, we describe the literature search strategy, inclusion criteria, and approach to data synthesis.

Literature Search Strategy

A comprehensive literature search was performed using PubMed and Google Scholar as the primary databases. The search covered publications from the earliest available records up to February 2025, ensuring inclusion of the most current research and clinical information. We focused on English-language literature to accommodate the authors' language proficiency. A combination of Medical Subject Headings (MeSH) terms and keywords was used to capture the breadth of the topic. Key search terms included "congenital eye disease", "pediatric ophthalmology", "congenital cataract", "retinopathy of prematurity", "retinal dystrophy", "macular dystrophy", "microphthalmia", "anophthalmia", and related terms, often paired with "etiology", "genetics", "diagnosis", or "management". These terms were used in various Boolean combinations to ensure comprehensive coverage (e.g., "congenital cataract AND genetics", "retinopathy of prematurity AND treatment", "microphthalmia OR anophthalmia AND children", etc.). In addition to database searching, we manually scanned the reference lists of key articles and relevant review papers to identify any additional publications missed in the initial search. Authoritative sources such as World Health Organization (WHO) fact sheets and ophthalmology textbooks or clinical guidelines were also consulted for foundational information and to cross-check data, especially for epidemiology and standard-of-care practices.

The search process was iterative and expansive. Whenever new relevant keywords or concepts emerged (for instance, names of specific genes, therapies, or classification systems relevant to these diseases), additional targeted searches were conducted. Throughout the search, duplicates were removed, and results were screened by title and abstract for relevance to pediatric congenital eye conditions. The final search was conducted on February 24, 2025, to include any early 2025 publications or updates. All searches and retrieval of articles were conducted by at least two authors working in parallel to broaden the yield and reduce the chance of missing important literature.

Inclusion and Exclusion Criteria

Given the broad, integrative aim of this narrative review, the inclusion criteria were intentionally expansive. We included peer-reviewed articles (original research studies, meta-analyses, and review articles) that addressed any aspect of the specified congenital eye diseases in children, including their epidemiology, genetic underpinnings, clinical presentation, diagnostic methods, or treatment approaches. Studies focusing on pediatric populations (from neonates through adolescents) with congenital or early-onset forms of the eye conditions were prioritized. We also included relevant clinical guidelines, consensus statements, and authoritative web resources (e.g., WHO and specialty ophthalmology organizations) that provided epidemiological data or standard definitions, in order to contextualize findings within current practice. Both clinical studies (such as case series, cohort studies, and clinical trials) and basic science research were considered if they offered insight into disease mechanisms (e.g., animal model studies elucidating the pathophysiology of ROP or genetic studies on inherited dystrophies were included). There was no strict restriction on study design; our goal was to capture a comprehensive picture of current knowledge on each condition.

We applied a few exclusion criteria to maintain focus. Articles were excluded if they did not specifically relate to congenital or early-onset disease (for instance, studies on adult-onset cataract or age-related macular degeneration were not included, unless they provided information transferable to congenital conditions). We also excluded publications that were purely anecdotal or very limited case reports unless they illustrated a unique facet of a congenital condition that was otherwise under-represented in larger studies. Non-English-language publications were excluded if no reliable English translation or summary was available. In cases where multiple sources provided duplicate information or data, preference was given to the most recent and comprehensive source or to the original source of novel findings (e.g., a seminal study describing a gene mutation was favored over later papers that cited it). To minimize bias in selection, at least two authors independently reviewed the titles and abstracts during the screening process. Any disagreements or uncertainties regarding inclusion were resolved through team discussion and consensus.

No formal quality appraisal (such as risk of bias scoring) was conducted on the included sources, consistent with the narrative review methodology. Our emphasis was on breadth and depth of coverage rather than on rating the level of evidence of each study. Nevertheless, we gave greater descriptive weight to high-impact studies, large sample investigations, and well-established evidence (such as randomized trial results or widely accepted guidelines) when synthesizing conclusions.

Data Extraction and Synthesis of Evidence

Data from the included literature were extracted in a narrative fashion by reading full texts and summarizing the key points relevant to our review objectives. Each author focused on one or more of the subtopics (congenital cataract, ROP, retinal dystrophies, macular dystrophies, microphthalmia/anophthalmia) to collate detailed notes on etiology, clinical features, diagnostic methods, and treatment options for that condition. We did not use a standardized data extraction form as one might in a systematic review; instead, we employed a thematic extraction approach. This involved identifying recurring themes and important findings in each article and organizing them under the review's key domains (genetic causes, clinical presentation, diagnostic tools, management strategies, and outcomes for each condition). The information was then cross-validated among the authors to ensure accuracy and consistency; for instance, if one author summarized the genetics of congenital cataracts, another author cross-checked this summary against the literature to confirm no major studies were overlooked.

The synthesis approach was entirely qualitative. We structured the Results (review findings) section of this article by disease category, dedicating separate subsections to each of the major conditions (cataracts, ROP, etc.). Within each subsection, we integrated findings from multiple sources to provide a cohesive overview. For example, data on the prevalence and causes of congenital cataracts from epidemiologic studies were combined with insights from genetic studies and reports on surgical outcomes to present a multifaceted understanding of that condition. We compared and contrasted findings across studies when relevant, highlighting, for instance, areas of consensus (such as the importance of early surgical intervention in bilateral congenital cataract) versus ongoing debates or inconsistencies (such as varying success rates of emerging gene therapies for retinal dystrophies). Throughout the synthesis, we aimed to maintain an objective tone, presenting both the strengths and limitations of current evidence. Where applicable, we noted gaps in the literature or emerging research directions (for instance, mentioning that while anti-vascular endothelial growth factor (anti-VEGF) therapy is advancing ROP management, long-term outcomes are still being studied).

All authors collaboratively reviewed the synthesized content to ensure that it accurately reflected the source material and that the narrative flow was logical and easy to follow. By organizing the review in this manner, we aimed to create a clear and comprehensive narrative that links etiology, diagnosis, and management for each congenital eye disease. This method of synthesis allows readers to understand each condition in depth and also facilitates the recognition of overarching themes in pediatric ocular health (such as the critical importance of early detection across all these conditions). We believe this narrative approach, underpinned by a systematic literature search and selection process, provides a robust and scholarly overview of congenital eye diseases in children suitable for a high-impact clinical audience.

Results

Retinal Dystrophies

Inherited retinal dystrophies represent a spectrum of congenital retinal disorders that lead to progressive vision loss [8]. They are associated with genetic mutations that result in abnormal protein products that affect several pathways that are important for vision, such as light perception, visual cycle, cellular respiration, development of photoreceptors, and retinal integrity [9]. This eventually causes dysfunction of the cone and rod photoreceptors or retinal pigment epithelium [10]. Infant respiratory distress syndrome (IRDS) includes retinitis pigmentosa (the most common form), Leber congenital amaurosis, choroideremia, and X-linked juvenile retinoschisis, which have many genetic and clinical variabilities [11]. These disorders are associated with hundreds of genes with autosomal dominant, autosomal recessive, X-linked, or mitochondrial inheritance patterns [12]. Recently, several studies have investigated the treatment options for infants with respiratory distress syndrome. Voretigene neparvovec, also known as Luxturna, is a gene therapy that uses an adeno-associated virus vector [13]. Vision is restored by introducing an intact copy of the RPE65 gene into the retinal cells of patients with low or no RPE65 protein [14]. Moreover, some studies have suggested that antisense oligonucleotides modify gene expression in rhodopsin mutation-induced autosomal dominant retinitis pigmentosa [15]. Other promising strategies include stem cell-based therapies such as photoreceptor replacement and transplantation of the retinal pigment epithelium [16]. By substituting healthy retinal cells produced from stem cells with damaged ones, these techniques may restore vision over the long term [17].

Congenital Cataract

Lens opacity or cataracts are caused by light scattering by either the disruption of the lens microarchitecture itself or large molecular weight protein clumps within lens cells [18]. One of the leading causes of childhood blindness globally is congenital cataracts, accounting for between 10% and 19.5% of the causes of blindness and impaired vision [19]. Most bilateral cataracts are caused by genetic changes, and autosomal dominant inheritance is the most common pattern, occurring in 44% of the families. Congenital illnesses, such as toxoplasma, syphilis, varicella-zoster, parvovirus B19, coxsackievirus, rubella, cytomegalovirus (CMV), and herpes simplex virus I and II (TORCH), are significant environmental factors that should be considered. In this age range, trauma and iatrogenic factors, including drug and radiation exposure, are also substantial but uncommon [20]. Congenital cataracts are categorized based on their morphology, cause, and development time. Some publications refer to a child's cataract diagnosis after three months of age as developing or infantile cataracts. Visual prognosis was established using this chronological classification. They also have various etiologies, including infectious, genetic, and systemic disorders, and ocular abnormalities. Morphology is another way to categorize congenital cataracts as complete, lamellar, nuclear, sutural, capsular (posterior/anterior), lenticonus, and membranous [21]. They can be found alone or in combination with glaucoma, anterior segment dysgenesis (ASD), microphthalmia, microcornea, and syndromic relationships [22]. Congenital cataracts tend to arise from mutations with severe functional consequences on the structure and function of the mutated protein. For transcription factors such as PITX3 and MAF, this may indicate the absence of a transcriptional activator at a critical point in lens development, failing proper lens structures and protein components [18]. Conventional eye examinations in newborns and infants are performed by neonatologists and pediatricians using the red reflex test. If this test revealed an eye abnormality, the patient was examined by an ophthalmologist [23]. It is critical to evaluate both eyes to determine whether a cataract is unilateral or bilateral. A difficulty with unilateral congenital cataracts is that, if left untreated, even a small cataract can result in permanent profound amblyopia in the affected eye [24]. An abnormal red reflex test after birth enables the rapid diagnosis of eye conditions [25]. Early detection is essential for achieving optimal visual function in congenital cataracts. The results of cataract surgeries performed in children have improved with the use of current procedures combining primary intraocular lens (IOL) implantation with microincision cataract removal [26]. Combining congenital cataract surgery with the rapid and effective treatment of amblyopia appears to be safe and beneficial [27]. Posterior capsule opacification, glaucoma, inflammation, and uveitis are among the complications that may arise during cataract surgery [28].

Microphthalmia

Microphthalmia is an uncommon birth condition; it is a reduction in the volume or size of the eye within the orbit [29]. It is characterized by corneal diameters less than 10 mm or anteroposterior globe diameters less than 20 mm [30,31]. Microphthalmia can develop either alone or in conjunction with another syndrome. Moreover, it can be simple or complex. An eye with simple microphthalmia is smaller, but has an intact anatomical structure. In contrast, microphthalmia is classified as complex when it is linked to anomalies in either the anterior or posterior segments [32]. The incidence of microphthalmia ranges from 1.5 to 19 per 10,000 newborns [33]. The etiology of microphthalmia is complex, with documented chromosomal, monogenic, and environmental factors. The genetic basis is not fully understood, but one of the most common mutations and perhaps the most clinically significant is the SOX2 gene [34]. Another contributing factor is the environment; the most compelling evidence seems to be related to infections acquired during pregnancy [35]. However, the mechanism of microphthalmia remains unclear. Studies suggested that postnatal ocular growth is crucial and hypothesized that decreased size of the optic cup, altered proteoglycans in the vitreous, low intraocular pressure, and abnormal growth factor production may all or partially affect the pathogenesis of simple microphthalmia, while inadequate production of secondary vitreous may result in complex microphthalmia. Multidisciplinary teams of ophthalmologists, pediatricians, and clinical geneticists are frequently responsible for patient management [36]. The goal of treatment is to stimulate both soft tissue and bone orbital growth simultaneously to maximize vision and improve aesthetic appearance. Surgical management may be the main treatment method for microphthalmia [37]. Conservative treatment with conformers was used to treat mild-to-moderate microphthalmia. Additional remodeling techniques for severe microphthalmia include soft tissue reconstruction and endo-orbital volume replacement using implants, expanders, and dermis-fat grafts. A study demonstrates that practically all patients tolerated a conformer and ocular prosthesis treatment satisfactorily [38].

ROP

ROP belongs to the group of premature and low birth weight infant eye diseases caused by the disorder of retinal vessels. It comes as the second leading cause of childhood blindness around the globe [39,40]. Risk factors include low gestational age, low birth weight, and prolonged exposure to supplementary oxygen [41]. Anemia, sepsis, metabolic factors, and necrotizing enterocolitis have also been associated with ROP [42-45]. The pathophysiology involves retinal angio-proliferation with failure of the central retinal vessels to reach the retinal periphery, leading to retinal hypoxia, neovascularization, vitreous hemorrhage, and, ultimately, vision loss [46,47]. Genetic and metabolic factors have also been implicated [48,49]. Timely detection and proper management are crucial for treating ROP. National treatment standards for ROP have been developed as a result of such studies as Cryotherapy for Retinopathy of Prematurity (CRYO-ROP) and Early Treatment for Retinopathy of Prematurity (ET-ROP) [50]. The intravitreal injection of bevacizumab has shown effectiveness in managing this condition [51].

Developmental Macular Dystrophy

The group of disorders known as developmental macular dystrophies are diverse in both their molecular genetic foundation and clinical features [52]. These conditions result in substantial loss of vision, usually due to progressive macular atrophy. Bilateral, comparatively symmetrical macular defects that severely compromise the central visual function are hallmarks of macular diseases. Even though fundus findings are mostly seen in the central retina, psychophysical, electrophysiological, or histological evidence of more extensive, generalized retinal involvement is present in the majority of macular dystrophies [53]. Based on the method of inheritance, hereditary macular dystrophies can be divided into three major groups: X-linked inheritance, autosomal recessive inheritance, and autosomal dominant inheritance. To date, the following seven genes have been linked to macular dystrophies: ABCA4, ELOVL4, PROML1, VMD2, peripherin/RDS, TIMP3, and XLRS [54]. Fluorescein angiography (FFA), electroretinography, electrooculography, ophthalmoscopy, and genetic testing are used to assess the existence of macular dystrophies and to distinguish between various etiologies [55].

Much less progress has been made in treating autosomal dominant disorders, such as the best disease and pattern dystrophy, which together account for a significant disease burden. In contrast to autosomal recessive Stargardt disease and X-linked recessive X-linked retinoschisis, numerous therapeutic approaches have been investigated. This is most likely because creating genetic therapeutics for autosomal dominant (AD) disease is more difficult than gene replacement, which can be utilized for autosomal recessive and X-linked disorders [56]. With new advancements in gene editing and slicing, this gap can be narrowed in the future. Depending on the genotype and phenotype of macular dystrophy, several therapies are available. Patients are recommended to avoid vitamin A supplements, minimize ultraviolet (UV) exposure to potentially decrease development, and receive sufficient social support in addition to supplying low-vision devices and assistive technology to help maximize their vision. The complement-mediated reaction to accumulating byproducts of the visual cycle is an example of pharmacotherapy that targets the visual cycle either directly or indirectly and is used to slow the progression of the disease [56]. A better understanding of the molecular genetics and mechanisms underlying inherited macular dystrophy has improved genetic counseling, disease classification, prognosis, and diagnosis, and the possibility of developing treatment regimens in the future [57].

Discussion

Congenital eye diseases, which are diseases the baby is born with due to the presence of certain genes, drugs, or alcohol exposure during pregnancy, can mean that the eye is structurally or functionally affected and displays vision impairment or other complications. These states are not very common and have an incidence of approximately 3.5-4.8 cases out of 10,000 live births in European countries [58]. Congenital cataract is a white opacification of the crystal lens and bilateral cataracts [18], which can cause severe damage if untreated and is the main cause of treatable childhood blindness worldwide. The other major eye condition is ROP [59], an eye disease affecting preterm infants [60]. These diseases can block the light and cause the image on the retina to be faulty, and performing the surgery at an early age is very important to have very good vision [61]. Retinal dystrophies are progressive eye diseases of the retina that have both clinical and genetic heterogeneity [20], risk of color blindness, peripheral vision abnormalities [62], and full blindness [63]. More than 270 genes are associated with different retinal dystrophies, some of which are associated with certain phenotypes [64]. Retinal dystrophies can be treated with or without other symptoms either as isolated events or as a result of other ocular pathologies. The retinal defects are in both symmetrical eyes, thus affecting similar vision in both eyes, and can be described as visuospatial but without any other cognitive impairment or positive visual symptoms such as air hunger [29,57,65,66]. There is no cure for macular dystrophy; however, gene therapy clinical trials are underway to correct abnormal genes in the retina. Microphthalmia is a rare condition associated with chromosomal disease, genetic syndromes, and intracranial abnormalities, occurring in one in 5000 live births [66]. It is best managed through a shared care approach between local ophthalmic and pediatric services and a specialist center [33,66]. Glasses are prescribed for significant refractive errors and for protection in children with only one sighted eye [33].

Comparative Analysis of Congenital Eye Diseases

Congenital eye diseases, including congenital cataracts, ROP, retinal dystrophies, macular dystrophies, and microphthalmia, demonstrate diverse etiological and clinical presentations. While congenital cataracts and ROP remain the leading causes of treatable childhood blindness, retinal and macular dystrophies present unique challenges due to their genetic heterogeneity and progressive nature [18,57]. Microphthalmia, though rare, is associated with significant ocular and systemic anomalies, necessitating a multidisciplinary approach to management [33].

Efficacy of Current Therapeutic Approaches

Surgical interventions, such as primary IOL implantation for congenital cataracts and cryotherapy for ROP, have substantially improved visual outcomes when performed early [24]. However, long-term complications like posterior capsule opacification and secondary glaucoma underscore the need for vigilant postoperative [27].

Innovative treatments, including gene therapy with voretigene neparvovec for retinal dystrophies, represent a paradigm shift in managing inherited ocular diseases [13]. Similarly, stem cell therapies show promise in restoring retinal function by replacing damaged photoreceptors, although long-term safety and efficacy data are still emerging [16].

Challenges in Management and Early Detection

Despite advancements, challenges in managing congenital eye diseases persist, particularly in low-resource settings. Limited access to genetic testing and advanced imaging technologies hampers early diagnosis, which is critical for conditions like ROP and congenital cataracts [1]. Socioeconomic factors further exacerbate disparities in care, with underserved populations experiencing delayed interventions and poorer visual outcomes [5].

Screening protocols, such as the red reflex test for newborns, have proven effective in detecting ocular abnormalities early [25]. However, integrating these protocols into national healthcare systems remains inconsistent, highlighting an area for policy development and public health initiatives.

Emerging Trends and Future Directions

The advent of personalized medicine offers new opportunities for managing genetically heterogeneous conditions like retinal and macular dystrophies. Advances in CRISPR-Cas9 technology and antisense oligonucleotides have the potential to modify pathogenic genetic variants, offering hope for conditions previously considered untreatable [15]. Additionally, research into pharmacological agents targeting the visual cycle and complement pathways may slow the progression of dystrophic diseases, enhancing the quality of life of affected children [56]. The integration of telemedicine and artificial intelligence (AI) in screening and monitoring could also improve access to specialized care, particularly in remote areas [47,67].

Implications for Clinical Practice and Research

For clinicians, these findings emphasize the need for multidisciplinary collaboration in managing congenital eye diseases. Ophthalmologists, pediatricians, geneticists, and rehabilitation specialists must work together to provide holistic care that addresses both the medical and developmental needs of affected children. The establishment of standardized guidelines for screening, diagnosis, and management is critical to reducing variability in care and improving outcomes. Research efforts should focus on longitudinal studies assessing the real-world effectiveness of novel therapies, as well as cost-effectiveness analyses to support their integration into clinical practice. Furthermore, exploring the psychosocial impact of visual impairment on children and their families could guide the development of targeted support services.

## Conclusions

Congenital eye diseases profoundly shape the visual future of children, presenting complex challenges that span genetic, clinical, and therapeutic landscapes. Despite breakthroughs in gene therapy and stem cell applications, disparities in access to these advancements persist, especially in underserved regions. This review reveals the intricate nature of congenital cataracts, ROP, retinal dystrophies, macular dystrophies, and microphthalmia, emphasizing the urgency for early diagnosis and tailored interventions. To transform care, a coordinated approach involving geneticists, ophthalmologists, pediatricians, and public health specialists is essential. Prioritizing research that bridges diagnostic gaps and advances personalized therapies will redefine outcomes for pediatric patients. By championing equitable healthcare strategies and leveraging innovative treatments, we can reshape the narrative of childhood blindness, steering towards a future where vision loss from congenital eye diseases is not a certainty but a preventable possibility.
